# Projected distribution patterns of *Alpinia officinarum* in China under future climate scenarios: insights from optimized Maxent and Biomod2 models

**DOI:** 10.3389/fpls.2025.1517060

**Published:** 2025-02-10

**Authors:** Yong Kang, Fei Lin, Junmei Yin, Yongjie Han, Min Zhu, Yuhua Guo, Fenling Tang, Yamei Li

**Affiliations:** ^1^ National Key Laboratory for Tropical Crop Breeding, Tropical Crops Genetic Resources Institute, Chinese Academy of Tropical Agricultural Sciences, Haikou, China; ^2^ Haikou Experiment Station, Chinese Academy of Tropical Agricultural Sciences, Haikou, China; ^3^ Sanya Research Institute, Chinese Academy of Tropical Agricultural Sciences, Sanya, China; ^4^ College of Horticulture Forestry Sciences, Huazhong Agricultural University, Wuhan, China; ^5^ College of Tropical Crops, Yunnan Agricultural University, Pu’er, China

**Keywords:** *Alpinia officinarum*, climate change, Maxent model, biomod2, species distribution prediction, suitability area

## Abstract

*Alpinia officinarum*, commonly known as Galangal, is not only widely used as a medicinal plant but also holds significant ornamental value in horticulture and landscape design due to its unique plant structure and floral aesthetics in China. This study evaluates the impact of current and future climate change scenarios (ssp126, ssp245, ssp370, and ssp585) on the suitable habitats for *A. officinarum* in China. A total of 73 reliable distribution points for *A. officinarum* were collected, and 11 key environmental variables were selected. The ENMeval package was used to optimize the Maxent model, and the potential suitable areas for *A. officinarum* were predicted in combination with Biomod2. The results show that the optimized Maxent model accurately predicted the potential distribution of *A. officinarum* in China. Under low emission scenarios (ssp126 and ssp245), the suitable habitat area increased and expanded towards higher latitudes. However, under high emission scenarios (ssp370 and ssp585), the suitable habitat area significantly decreased, with the species distribution range shrinking by approximately 3.7% and 19.8%, respectively. Through Multivariate environmental similarity surface (MESS) and most dissimilar variable (MoD) analyses revealed that increased climate variability under high emission scenarios, especially in ssp585, led to large-scale habitat contraction due to rising temperatures and unstable precipitation patterns. Changes in the center of suitability location showed that the current center of *A. officinarum*’s suitable habitat is located in Guangxi, China. Under low emission scenarios, the center of suitability gradually shifts northwest, while under high emission scenarios, this shift becomes more pronounced. These findings provide a scientific basis for the conservation of *A. officinarum* germplasm resources and the management strategies in response to climate change.

## 1 Introduction

Global climate change is one of the most critical environmental challenges of the 21st century. Over the past 100 years, global temperatures have gradually increased, with the rate of warming accelerating over the past 30 years, profoundly affecting ecosystems and biodiversity (Khan and Verma, 2022; Huang et al., 2024). Climate change directly affects plant growth and distribution, particularly as shifts in temperature and precipitation patterns drive plant habitats to gradually migrate towards higher latitudes and elevations in response to the increasing environmental pressures (Heckathorn et al., 2020; Pareek et al., 2020). Climate change not only affects the biogeographical patterns of plants but may also significantly alter ecosystem services (Camille and Hanley, 2015).

In recent years, changes in plant distribution patterns have become a research focus in the fields of ecology and conservation biology (Gurung et al., 2024). Research has shown that climate change may lead to habitat contraction or migration for plant species, thereby threatening species diversity and the stability of ecosystems (Tang et al., 2021). Particularly, species with high ecological sensitivity and narrow distribution ranges are more vulnerable to adverse effects in the face of climate change (Porfirio et al., 2014). Global warming has caused significant changes in plant distribution in tropical and temperate regions, with extreme weather events such as droughts and floods further intensifying these shifts (Qazi et al., 2022). Climate change has also altered ecosystem functions and services. For example, the decline in plant diversity in certain regions has led to reduced ecosystem productivity and stability, which in turn weakens the ability of ecosystem to adapt to climate change and increases its ecological risks (Correia and Lopes, 2023). Therefore, understanding the mechanisms by which plants respond to climate change, especially predicting future distribution pattern changes based on global climate models, is crucial for developing effective ecological conservation strategies (Parmesan and Hanley, 2015).

Species Distribution Models (SDMs) have become one of the primary tools for studying the impact of climate change on plant distribution. These models integrate known species distribution data with environmental variables (such as temperature, precipitation, and soil type) to effectively predict the potential suitable habitats for plants under different future climate scenarios (Srivastava et al., 2019). Among these models, the Maximum Entropy Model (Maxent) was chosen for its ability to handle small sample sizes while maintaining high predictive accuracy Maxent (Xu et al., 2024), is especially suitable for species with limited distribution data, such as *A. officinarum*. Its global application has demonstrated that Maxent can provide reliable predictive results even in cases of limited sample data, making it particularly suitable for assessing the potential habitat ranges of plant species (Chahouki and Sahragard, 2016; Wang et al., 2024c). In addition, the Biomod2 package integrates multiple models, such as Generalized Linear Models (GLM), Generalized Additive Models (GAM), Artificial Neural Network (ANN), Classification Tree Analysis (CTA), Flexible Discriminant Analysis (FDA), Surface Range Envelope (SRE) and Random Forest (RF), to optimize predictive results and improve accuracy (Wang et al., 2024a). This integration enhances the robustness and reliability of predictions, generating more reliable and optimized results, and making it a valuable tool for this study (Thuiller et al., 2009; Zhao et al., 2021). The Maxent and Biomod2 provide a powerful multi-model approach for studying how plants respond to future climate change, aiding scientists in developing effective ecological conservation and resource management strategies (Huang et al., 2023; Gu et al., 2024).

*Alpinia officinarum* is a perennial herbaceous plant widely distributed in the humid, low-altitude regions of southern China. It holds significant medicinal and ornamental value, along with substantial market demand (FOC, E.C.o, 1981). Since the beginning of the 21st century, wild populations of *A. officinarum* have been critically endangered, with most commercially available products now derived from cultivated sources. Xuwen County in Guangdong Province is one of the key production areas for this species. *A. officinarum* is native to tropical and subtropical regions and thrives in warm, humid climates. It is drought-tolerant but sensitive to waterlogging and frost. The species grows optimally within a temperature range of 38.8°C (maximum) to 2.2°C (minimum) and annual precipitation levels between 1,100 and 1,803 mm. During its seedling stage, *A. officinarum* is not adapted to strong sunlight and requires some degree of canopy cover, but mature plants can tolerate higher light intensities. While the species is not particularly demanding in terms of soil, it thrives best in deep, loose, fertile, humus-rich soils with slightly acidic to acidic pH. In recent years, the suitability of its habitat has been severely impacted by both anthropogenic activities and climate change, especially with increasing temperatures, altered precipitation patterns, and a rise in extreme climate events (Yang et al., 2012). Therefore, studying the habitat characteristics, climate sensitivity, and adaptive responses of *A. officinarum* is essential for evaluating its vulnerability in the context of ongoing climate change. With global warming and the increasing frequency of extreme climate events, the natural habitat of *A. officinarum* may face risks of contraction and migration, posing a threat to the long-term stability of its populations. Existing research has primarily focused on the physiological and ecological characteristics as well as the chemical composition of *A. officinarum* (Van et al., 2021; Youn et al., 2024), but there is a lack of systematic research on its distribution dynamics under future climate change scenarios. Understanding and predicting the adjustment trends of *A. officinarum’s* suitable habitat under climate change is crucial for developing conservation plans and ensuring its sustainable utilization. We can assess the suitable habitat distribution of *A. officinarum* under different climate scenarios and identify potential areas of habitat expansion and contraction by applying species distribution models (Roy et al., 2022). This provides a scientific basis for the conservation of *A. officinarum* germplasm resources and its sustainable utilization.

Climate change will continue to profoundly impact the survival environments and geographical distribution patterns of plants (Kosanic et al., 2018). Conducting adaptive research on plants like *A. officinarum*, which hold significant ecological and economic value, not only aids in understanding the long-term effects of climate change on biodiversity but also provides scientific support for future ecological conservation and resource management strategies. To effectively analyze the dynamic changes in suitable habitats for *A. officinarum* under various climate change scenarios, this study employed the ENMeval package to optimize Maxent model parameters and used Biomod2 in combination with Maxent to compare the predictive performance of different algorithms. Finally, the optimized models were then applied to forecast the potential distribution of *A. officinarum* under current and future climate conditions. The main objectives of this study are: 1) To predict the trends in suitable habitat changes of *A. officinarum* under different climate scenarios based on Maxent and Biomod2 models; 2) To assess the impact of climatic factors on the habitat of *A. officinarum* using MESS and MoD analyses, identifying potential areas of habitat expansion or contraction; 3) To predict the migration trends of habitat centers under future climate scenarios, providing scientific support for the conservation and sustainable use of *A. officinarum*. Maxent.

## 2 Materials and methods

### 2.1 Distribution area

**Figure 1:**
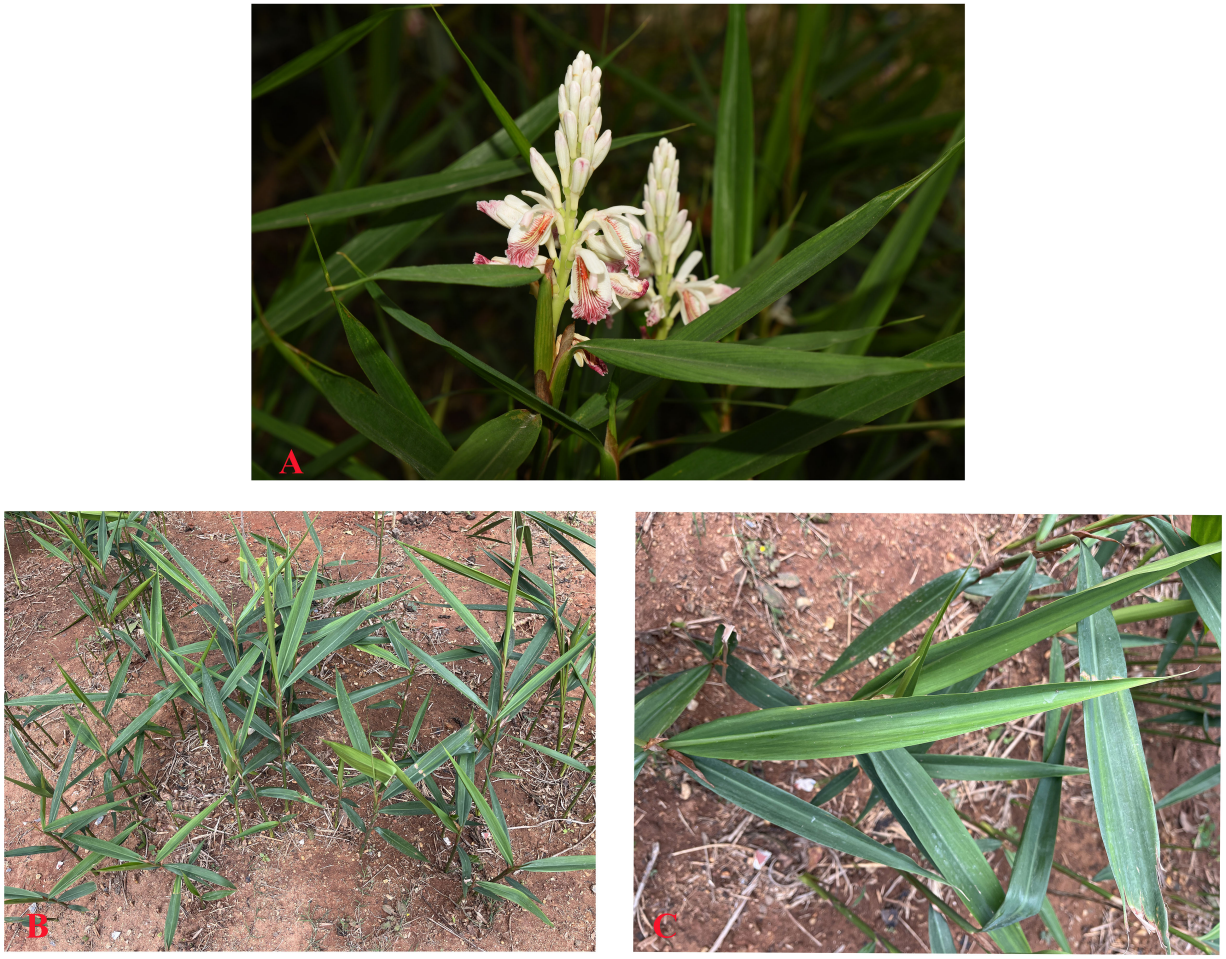
*Alpinia officinarum.* **(A)** Flower, **(B)** Whole plant, **(C)** Leaf.

*A. officinarum*, a perennial herbaceous plant in the Zingiberaceae family, is one of the widely distributed representative species of the ginger family in southern China (**Figure 1**). It primarily grows in regions such as Guangdong, Guangxi, and Hainan, commonly found in low-altitude mountainous areas, forest edges, and moist areas along rivers. The species exhibits strong ecological adaptability and environmental tolerance. In this study, by analyzing the geographic data of its growing environment and distribution range, we identified that the primary distribution area of *A. officinarum* is located between 100°51’36” to 117°10’48” E and 18°30’00” to 27°23’60” N.

### 2.2 Collection and screening of sample data

A total of 84 distribution records of *A. officinarum* were collected by reviewing published literature and retrieving data from global biodiversity information repositories, including the Global Biodiversity Information Facility (GBIF, http://www.gbif.org), Chinese Virtual Herbarium (CVH, http://www.cvh.org.cn), and Chinese Field Herbarium (CFH, http://www.cfh.ac.cn). During the data processing, the collected distribution records were rigorously filtered to exclude points with unclear geographic information, duplicates, or outliers. After careful organization and validation, 73 reliable natural distribution points of *A. officinarum* within China were confirmed for use in the study.

### 2.3 Screening of environmental variables

In total, 28 environmental factors were selected for the study, including 19 bioclimatic factors, 1 elevation factor, and 8 soil factors. The bioclimatic data used in this study includes 19 key climatic factors from the current period (1970-2000), as well as environmental projection data for the future periods of the 2050s (2041-2060) and the 2090s (2081-2100). These climate data and the 1 elevation factor were obtained from the global climate database WorldClim (http://www.worldclim.org), with a spatial resolution of 30 arc-seconds (approximately 1 kilometer), which accurately reflects regional climate change characteristics. For future climate data, this study utilized the CMCC-ESM2 model, which is part of the Coupled Model Intercomparison Project Phase 6 (CMIP6). CMCC-ESM2 is particularly suitable for simulating climate change in tropical and subtropical regions, including China and surrounding areas, where it has demonstrated high accuracy in climate predictions. The model effectively captures regional climate patterns, particularly changes in temperature and precipitation. CMIP6 data provides climate projections for four different Shared Socioeconomic Pathways, including a sustainable development scenario (ssp126), a moderate development scenario (ssp245), a medium-high emission scenario (ssp370), and a traditional high emission scenario (ssp585). Compared to the previous generation CMIP5 data, the SSP scenarios in CMIP6 offer higher precision and differentiation, allowing for better integration of socio-economic factors in regional predictions, thus providing a more scientific basis for future climate change assessments. The 8 soil factors were obtained from the Harmonized World Soil Database (HWSD) of the Food and Agriculture Organization (FAO) (http://www.fao.org/faostat/en/#data), while the vector maps of China were obtained from the Ministry of Natural Resources of China (http://www.mnr.gov.cn/). The environmental variables selected in the preliminary stage of this study are shown in **Table 1**.

**Table 1:** Environmental variables.

Factor	Description	Unit
bio1	Annual Mean Temperature	°C
bio2	Mean Diurnal Range (Mean of monthly (max temp - min temp))	°C
bio3	Isothermality (BIO2/BIO7) (×100)	%
bio4	Temperature Seasonality (standard deviation ×100)	°C
bio5	Max Temperature of Warmest Month	°C
bio6	Min Temperature of Coldest Month	°C
bio7	Temperature Annual Range (BIO5-BIO6)	°C
bio8	Mean Temperature of Wettest Quarter	°C
bio9	Mean Temperature of Driest Quarter	°C
bio10	Mean Temperature of Warmest Quarter	°C
bio11	Mean Temperature of Coldest Quarter	°C
bio12	Annual Precipitation	mm
bio13	Precipitation of Wettest Month	mm
bio14	Precipitation of Driest Month	mm
bio15	Precipitation Seasonality (Coefficient of Variation)	%
bio16	Precipitation of Wettest Quarter	mm
bio17	Precipitation of Driest Quarter	mm
bio18	Precipitation of Warmest Quarter	mm
bio19	Precipitation of Coldest Quarter	mm
Elevation	Height above sea level	meters (m)
Aspect	Slope direction	degrees (°)
Slope	Terrain steepness	degrees (°)
t_clay	Clay content	%
t_gravel	Gravel amount	%
t_oc	Soil fertility indicator	%
t_pH	Soil acidity/alkalinity	pH value
t_ref	Light reflection	%
t_sand	Sand content	%

### 2.4 Model calibration

In this study, the ENMeval and biomod2 packages in R were employed to optimize and construct the environmental distribution models. ENMeval helps optimize the key parameter settings of the Maxent (v.3.4.4) model, thereby enhancing the predictive accuracy of model (Bai et al., 2024). Through the ENMeval package, two Maxent parameters were adjusted: the regularization multiplier (RM) and the feature combination (FC). Maxent offers five feature types: linear (L), quadratic (Q), hinge (H), product (P), and threshold (T). By default, the RM parameter is set to 1, and the FC combination is LQHPT. To optimize model performance, the RM parameter was adjusted between 0.5 and 4, in increments of 0.5. For feature combinations, six different settings were tested, including L, LQ, H, LQH, LQHP, and LQHPT. Parameter combinations were tested using ENMeval, and the best combination was selected based on the delta value of the corrected Akaike Information Criterion (AICc). The AIC is used to assess model complexity and fit, and the model selection was based on the combination with the lowest AICc delta value (delta.AICc=0), indicating the optimal balance between model complexity and goodness of fit (Chen et al., 2024). Additionally, the study referenced the mean area under the curve (avg.diff.AUC) of the optimized model and the average 10% test omission rate (avg.test.or10pct) to evaluate the fit of model to the local species distribution points (Muscarella et al., 2014).

The second step involved using the Biomod2 package in R to create the models. This study applied eight algorithms: Artificial Neural Network (ANN), Classification Tree Analysis (CTA), Flexible Discriminant Analysis (FDA), Generalized Additive Model (GAM), Generalized Linear Model (GLM), Random Forest (RF), Surface Range Envelope (SRE), and Maximum Entropy (Maxent). These models were chosen for their ability to handle different types of ecological data and their strengths in modeling species distributions. Each algorithm was selected for its unique strengths, including capturing non-linear relationships, handling large datasets, and providing accurate predictions for species distributions in varying environmental (Bi et al., 2022; Wang et al., 2023b). Except for Maxent, all other models used the default model settings in Biomod2. During the modeling process, 75% of the 73 distribution points for *A. officinarum* were randomly selected as training data, with the remaining 25% designated as test data. To better simulate actual distribution and minimize spatial bias, 1,000 pseudo-absence points were randomly selected. The selection of 1,000 pseudo-absence points was crucial to ensure a balanced representation of both suitable and unsuitable environmental conditions, which helps reduce spatial bias, particularly in areas with sparse distribution data. This approach enhances the model’s reliability by providing sufficient data for distinguishing between suitable and unsuitable habitats. The model construction was repeated 10 times to further minimize spatial bias and account for potential variability, ensuring more robust and accurate predictions.

Using the optimized model, simulations were performed to predict the suitable areas for *A. officinarum* under both current and future climate conditions. The Area Under the Curve (AUC) was used to assess the accuracy of the Maxent predictions, with AUC values ranging from 0 to 1.0. The larger the AUC value, the more accurate the prediction. An AUC value between 0.5 and 0.7 indicates poor predictive performance, 0.8 to 0.9 indicates good performance, and values between 0.9 and 1.0 represent excellent predictive accuracy (Phillips et al., 2006; Hao et al., 2020). In this study, ArcGIS 10.2 was used to convert the model outputs, and the natural breaks method was applied to classify the results into habitat suitability gradients. These were divided into four categories: 0-0.10 indicating unsuitable areas, 0.10-0.30 as low suitability areas, 0.30-0.60 as moderately suitable areas, and 0.60-1.0 representing highly suitable areas. The area of suitable habitat under different climate scenarios was calculated using ArcGIS 10.2.

### 2.5 Spatial pattern changes in suitable areas for *A. officinarum*

Spatial units with a species presence probability value ≥0.30 were considered suitable areas for *A. officinarum*, while units with a probability value <0.30 were classified as unsuitable. Based on this classification, presence/absence (0,1) matrices were established to represent the potential geographic distribution of *A. officinarum* under both current and future climate change scenarios. Suitable areas were assigned a value of 1 (presence), while unsuitable areas were assigned a value of 0 (absence). Using these matrices, the spatial pattern changes of suitable areas for *A. officinarum* under current and future climate scenarios were further analyzed. Four types of suitability changes were defined: newly suitable areas, lost suitable areas, retained suitable areas, and unsuitable areas. The changes in the area of suitability were calculated based on the current and projected future suitable habitat areas. The spatial pattern changes in potential suitable areas under current and future climate conditions were defined as follows: a matrix value change from 0→1 indicates newly suitable areas, 1→0 indicates lost suitable areas, 1→1 represents retained suitable areas, and 0→0 represents unsuitable areas (Huang et al., 2019; Zhang et al., 2022).

### 2.6 Multivariate environmental similarity surface and most dissimilar variable analyses

The Multivariate Environmental Similarity Surface (MESS) represents the degree of similarity between a set of predictor variables (V1, V2, Vi…) and a reference set of points. In the reference layer, *mini* and *maxi* refer to the minimum and maximum values of the environmental variable *Vi*, respectively, and *pi* is the value of the environmental variable *Vi* at point *P* in the reference layer during a specific period. The variable *fi* represents the percentage of points in the study area where the environmental variable *Vi* is less than *pi*. When *fi* = 0, the MESS value is calculated as 100 × (pi - mini)/(maxi - mini); when 0 < *fi* ≤ 50, the MESS value is 2fi; when 50 < *fi* < 100, the MESS value is 2(100 - *fi*); and when *fi* = 100, the MESS value is calculated as 100 × (maxi - pi)/(maxi - mini). The MESS value for point *P* is the minimum similarity score across all environmental variables, also known as the “Most Dissimilar Variable” (MoD) (Clarke, 2013). A negative MESS value indicates that at least one variable falls outside the environmental range observed in the reference point set for a given period, a condition known as a climate anomaly. This means that the environmental conditions at point P are beyond the ecological adaptability of species, highlighting potential regions where the species may struggle to survive under future climate conditions. A MESS value of 100 indicates that the climate environment is fully consistent with the reference layer, representing normal climate conditions. These thresholds for climate anomalies play a crucial role in understanding the environmental limits of species’ habitats, allowing researchers to identify areas where species may face ecological stress or habitat loss due to climate change. The Maxent (v.3.4.4) software tool *density.tool.novel* was used to compute MESS and MoD values (Elith et al., 2010; Wang et al., 2024b).

### 2.7 Migration analysis of suitable habitat center point

In this study, ArcGIS 10.2 software was used to calculate the center point of the current and future suitable areas for *A. officinarum* and analyze the trends of change. The suitable habitat of *A. officinarum* was treated as a single entity, simplified into a central point, and the movement of this center was used to reflect changes in the size and direction of the suitable habitat. The migration distance of suitable areas in terms of latitude and longitude can be assessed by analyzing the center of *A. officinarum* under different periods and climate conditions (Jin et al., 2023).

## 3 Results

### 3.1 Correlation and multicollinearity analysis of environmental variables

**Figure 2:**
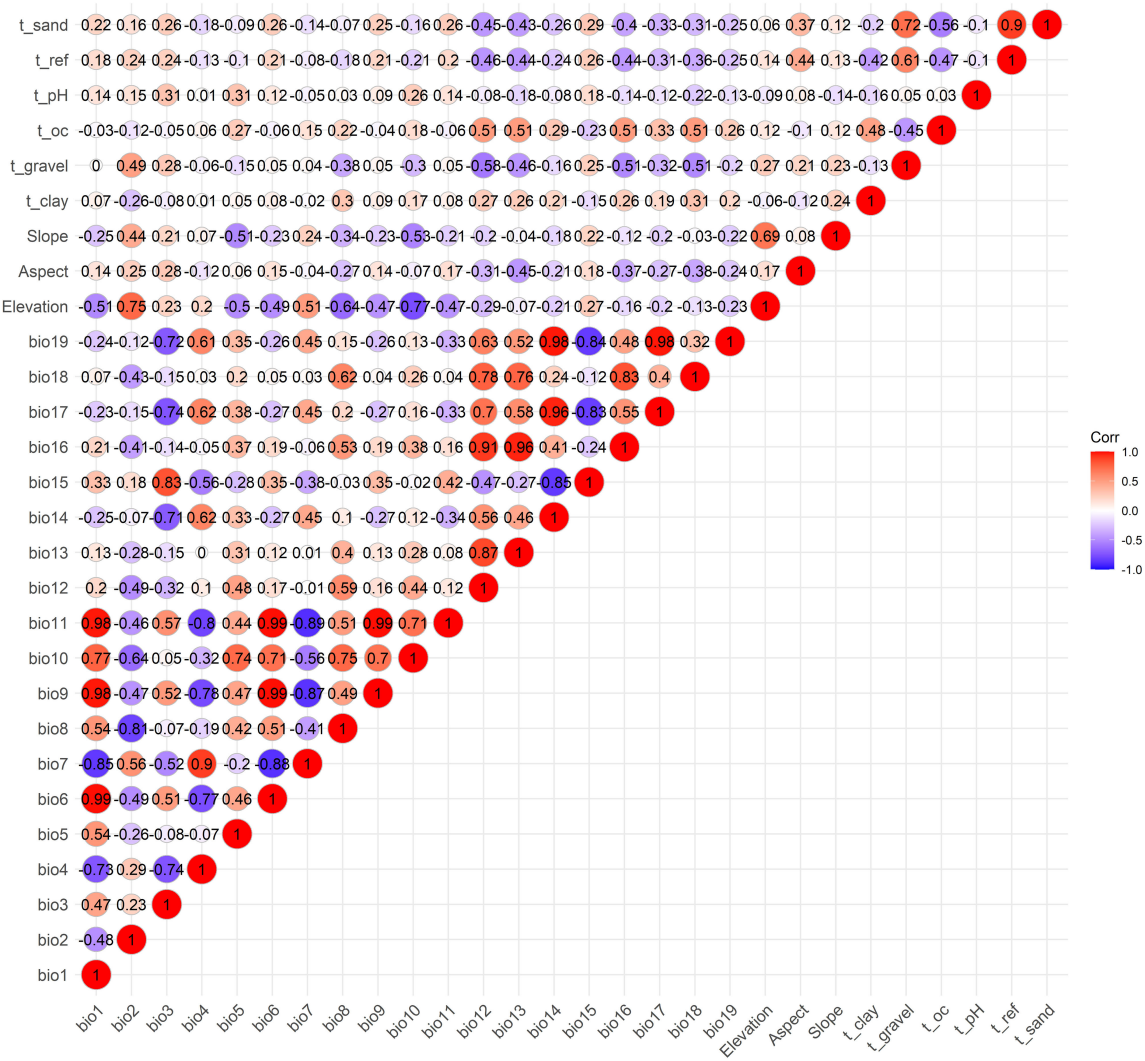
Spearman correlation analysis of the 28 environmental variables.

Spearman correlation analysis was performed on 28 environmental factors using R 4.3.3, and 11 factors with a correlation coefficient <0.7 were selected for further analysis. These included 4 climatic variables (bio5, bio7, bio15, bio18), 3 topographical variables (Elevation, Aspect, Slope), and 4 soil variables (t_clay, t_gravel, t_oc and t_pH) (**Figure 2**). Using bio5 as the independent variable, linear regression and multicollinearity analysis (Variance Inflation Factor, VIF) were conducted with the other 10 environmental factors as dependent variables. The results indicated no multicollinearity among the 11 environmental factors (VIF < 10) (**Table 2**). Therefore, 11 environmental factors including bio5, bio7, bio15, bio18, Elevation, Aspect, Slope, t_clay, t_gravel, t_oc and t_pH, were ultimately selected for further analysis in this study.

**Table 2:** VIF values between bio5 and other environmental factors.

Variable	bio7	bio15	bio18	Elevation	Aspect	Slope	t_clay	t_gravel	t_oc	t_pH
VIF	1.94	2.64	3.12	2.40	1.44	5.97	2.72	1.94	3.81	4.19

### 3.2 Model optimization

**Figure 3:**
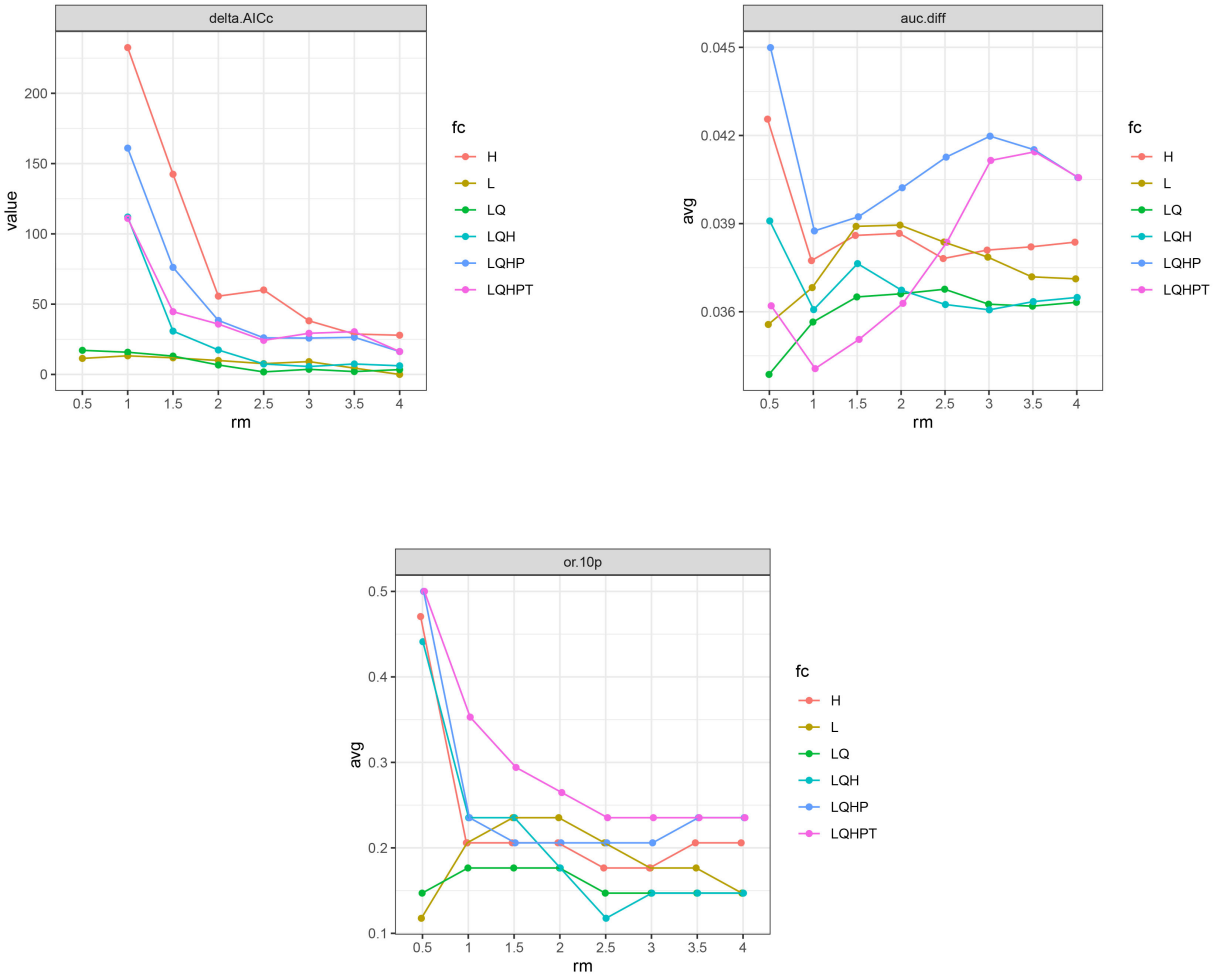
Maxent model optimization for delta.AICc, avg.diff.AUC, and avg.test.or10pct.

In this study, based on 73 distribution points of *A. officinarum* and 11 environmental variables, the Maxent model was optimized using the ENMeval package in R 4.3.3 to simulate the potential habitat distribution of *A. officinarum*. Under the default parameter settings (RM=1, FC =LQHPT), the analysis results showed a delta AICc of 111.06, an average AUC difference (auc.avg.diff) of 0.034, an AUC difference standard deviation (auc.diff.sd) of 0.179, and an average 10% test omission rate (or.10p.avg) of 0.353. After optimization with ENMeval, the Maxent model parameters were adjusted to RM=4.0 and FC=L. In this case, the delta AICc was reduced to 0, indicating the optimal balance between model complexity and fit, ensuring accurate predictions. Additionally, the auc.avg.diff was 0.037, the auc.diff.sd was 0.160, and the or.10p.avg decreased to 0.147, demonstrating enhanced performance (**Figure 3**). Overall, the optimized parameter settings significantly improved the fit of model and enhanced its ability to predict species migration. Therefore, RM=4.0 and FC=L were adopted as the final parameter settings for this study.

### 3.3 Response curve analysis

**Figure 4:**
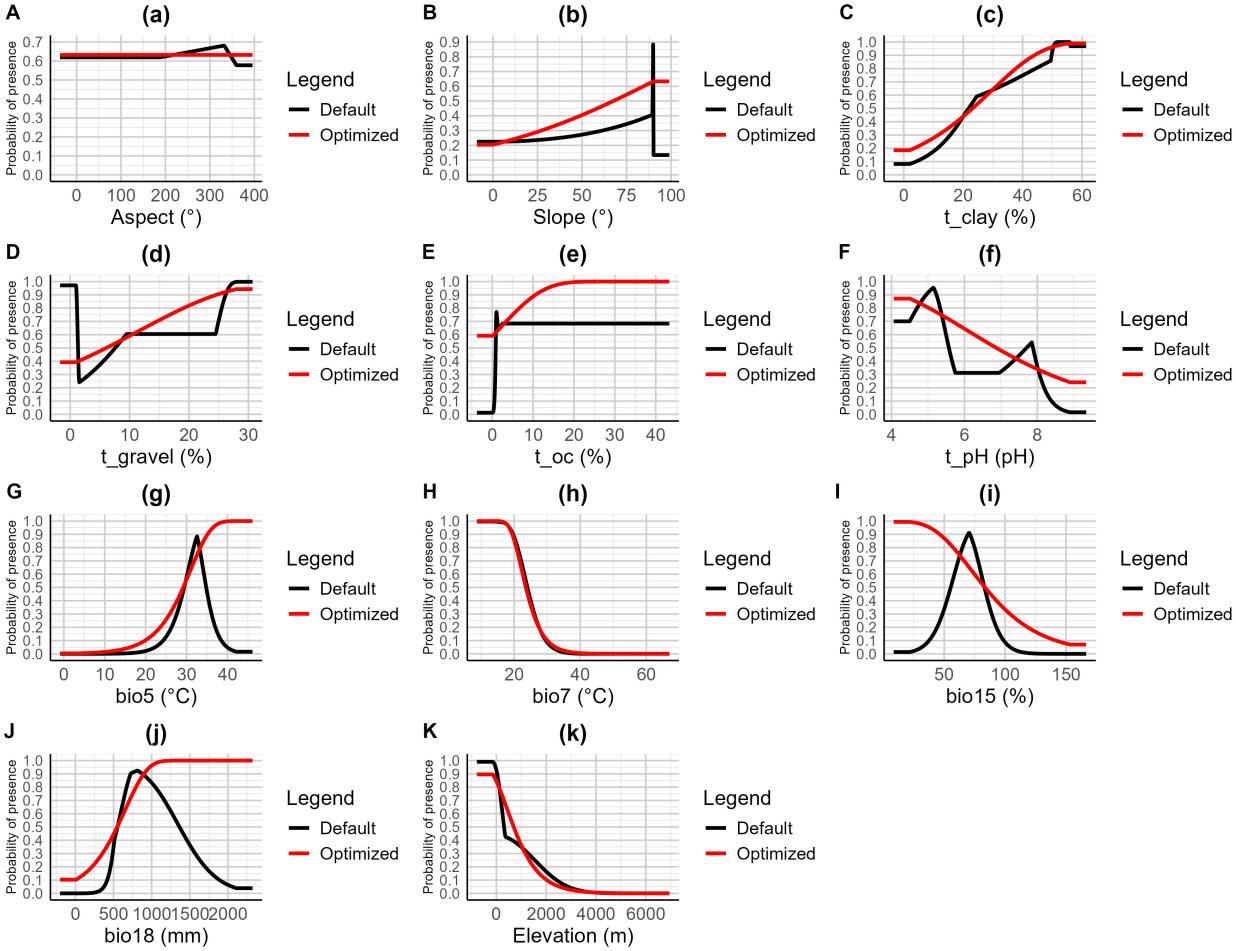
Response curves of 11 environmental factors in the habitat distribution model of *A. officinarum*. **(A)** Aspect, **(B)** Slope, **(C)** t_clay, **(D)** t_gravel, **(E)** t_oc, **(F)** t_pH, **(G)** bio5, **(H)** bio7, **(I)** bio15, **(J)** bio18, **(K)** Elevation.

The occurrence probability curves of the optimized model under various environmental factors exhibited a more stable trend compared to the default model (**Figure 4**). For variables such as Slope and Soil Organic Carbon (t_oc), the optimized model showed a gradual increase in probability as the factor value increased, stabilizing at higher values, whereas the default model exhibited sharp fluctuations in certain ranges. In the case of the annual maximum temperature (bio5) and the seasonality of annual mean temperature (bio7), the optimized model showed a continuous upward trend as temperature increased, displaying a smoother pattern compared to the steep rises observed in the default model in certain intervals. Additionally, for variables like Soil pH (t_pH) and Precipitation Seasonality (bio15), the optimized model demonstrated more linear and uniform changes, reducing the multiple fluctuations seen in the default model. As a result, the response curves of the optimized model were more consistent across most environmental factors.

### 3.4 Potential suitable habitat for *A. officinarum* under current climate conditions

**Figure 5:**
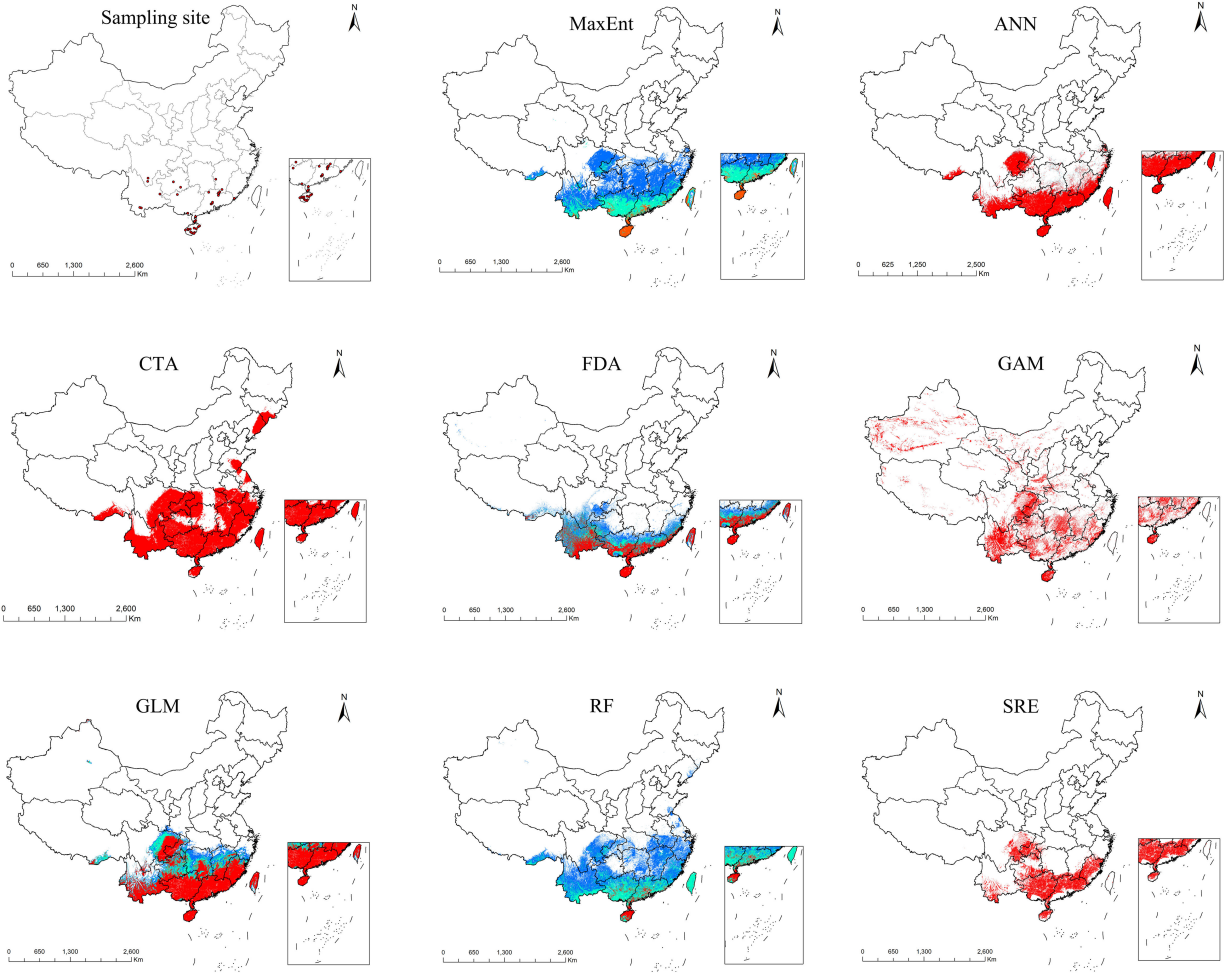
Spatial distribution records of *A. officinarum* and predicted potential geographic distribution by eight species distribution models.

The eight species distribution models generated by biomod2 show some variation in the predicted distribution of *A. officinarum* (**Figure 5**). Among these, the optimized Maxent model exhibited the best fit with the actual distribution points. Therefore, the Maxent model optimized using the ENMeval function was selected for the final modeling. Under current climate conditions, the highly suitable habitat for *A. officinarum* covers an area of 8.71×10^4^ km², accounting for 0.91% of the land area of China; the moderately suitable habitat covers 43.01×10^4^ km², accounting for 4.48%. And the generally suitable habitat covers 105.98×10^4^ km², accounting for 11.04% of the land area of China. In total, the suitable habitat area for *A. officinarum* is 157.70×10^4^ km², representing 16.43% of China total area. According to the results shown in **Figure 5**, the total suitable habitat for *A. officinarum* in China is primarily distributed in the river basins south of the Yangtze River, including provinces such as Guangxi, Guangdong, and Hainan.

### 3.5 Dynamic changes in suitable habitat for *A. officinarum* under different climate scenarios

The suitable habitat area for *A. officinarum* shows significant fluctuations and changes over time under different climate scenarios. The current total suitable area is 157.7×10^4^ km². Between 2041-2060 period, under low-emission scenarios (ssp126 and ssp245), the suitable area increases to 169.7×10^4^ km² and 164.7×10^4^ km², with an increase of 4.4% to 7.6%. In contrast, under high-emission scenarios (ssp370 and ssp585), the suitable area decreases to 151.9×10^4^ km² and 126.5×10^4^ km², representing a reduction of 3.7% to 19.8%. By 2081-2100 period, the suitable area under ssp245 reaches a peak of 174.6×10^4^ km², an increase of 10.7% compared to the present. However, under ssp585, the area decreases to 169.6×10^4^ km², reflecting a 7.5% increase compared to the current area (**Table 3**).

**Table 3:** Area changes of suitable habitats under different climate scenarios.

Period	Climate scenario	Unsuitable area (* 10^4^ km^2^)	Low-grade suitable area (* 10^4^ km^2^)	Moderately suitable area (* 10^4^ km^2^)	Highly suitable area (* 10^4^ km^2^)	Total suitable area (* 10^4^ km^2^)
current	–	802.30	105.98	43.01	8.71	157.70
2041-2060	ssp126	790.26	111.71	35.82	22.21	169.74
	ssp245	795.28	103.44	38.15	23.13	164.72
	ssp370	808.14	100.26	33.27	18.33	151.86
	ssp585	833.49	80.93	32.04	13.54	126.51
2081-2100	ssp126	809.79	97.50	34.27	18.44	150.21
	ssp245	785.37	112.03	39.30	23.30	174.63
	ssp370	812.46	95.78	34.23	17.53	147.54
	ssp585	790.41	108.24	37.73	23.62	169.59

The expansion and contraction of suitable areas exhibit marked variation. Between 2041-2060 period, under ssp126 and ssp245 scenarios, the expansion areas are larger, with 11.90×10^4^ km² and 7.59×10^4^ km², respectively, while contraction areas are minimal, ranging from 0.22×10^4^ km² to 0.81×10^4^ km². Under high-emission scenarios such as ssp370 and ssp585, the expansion areas are significantly reduced to 3.61×10^4^ km² and 1.96×10^4^ km², while contraction areas increase sharply to 9.49×10^4^ km² to 32.32×10^4^ km², indicating rapid expansion of unsuitable areas in high-emission scenarios.

**Figure 6:**
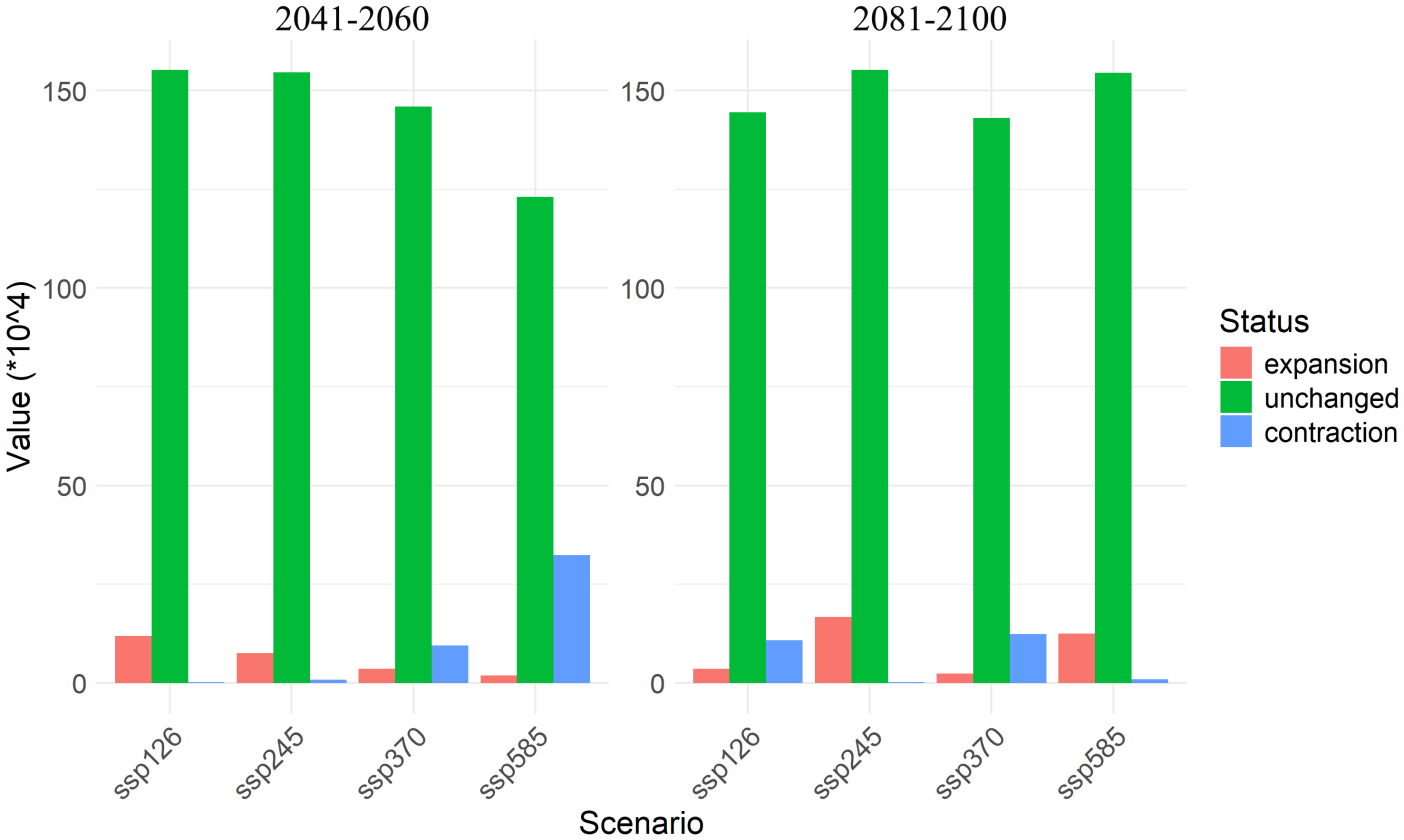
Expansion, contraction, and unchanged areas of *A. officinarum* suitable habitats under different climate scenarios.

By 2081-2100 period, the expansion area under the ssp126 scenario decreases to 3.54 × 10^4^ km², with contraction areas increasing to 10.87×10^4^ km². Under ssp245, the expansion area reaches its maximum of 16.76×10^4^ km², with the smallest contraction area of only 0.25×10^4^ km². However, under ssp370 and ssp585, the expansion areas decrease to 2.34×10^4^ km² and 12.53×10^4^ km², respectively, while contraction areas rise to 10.87×10^4^ km² to 12.38×10^4^ km² (**Figure 6**).

### 3.6 Analyzed the multivariate environmental similarity surface and most dissimilar variable

**Figure 7:**
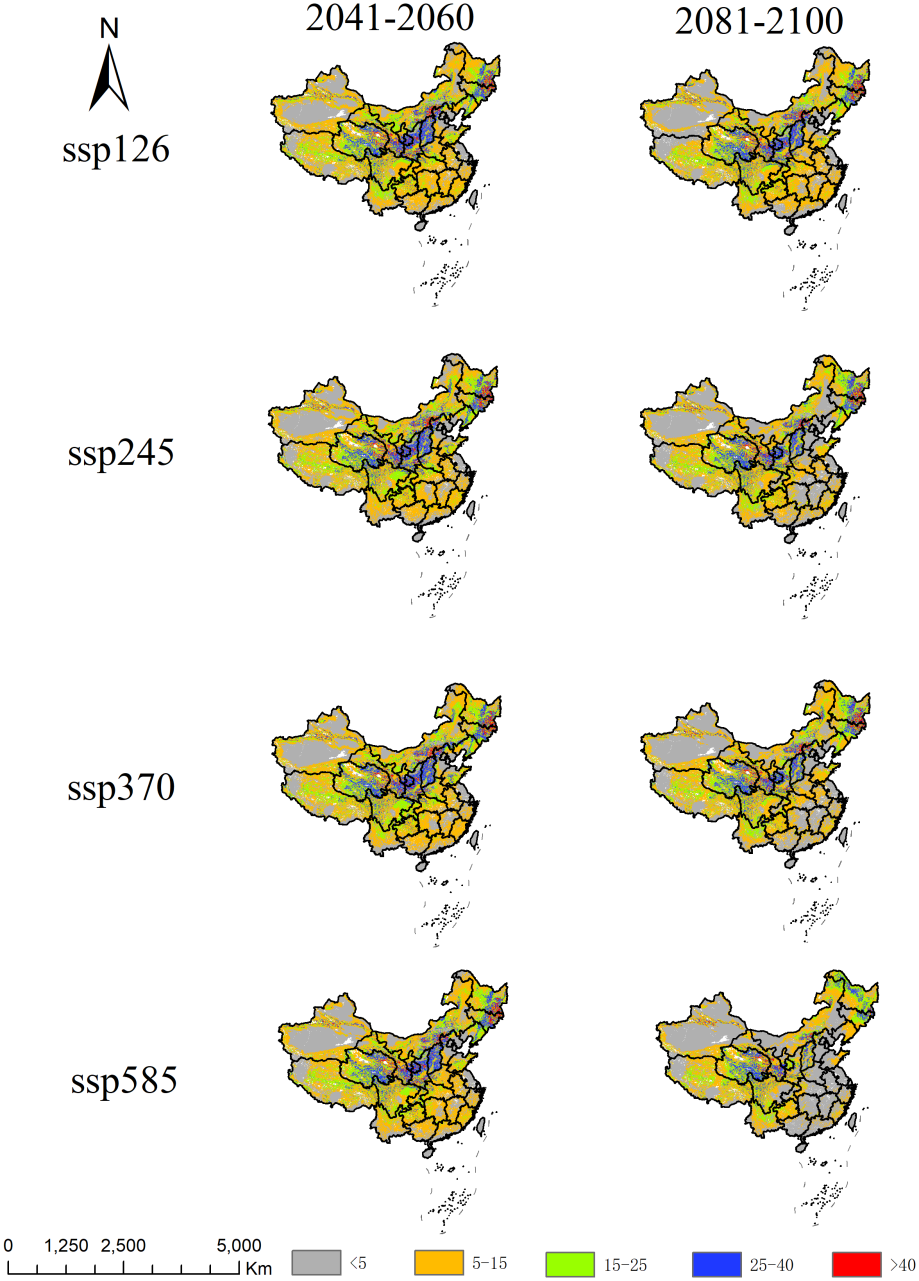
Analysis of multivariate environmental similarity surface for *A. officinarum* suitable habitats under different climate scenarios.

This study analyzed the multivariate environmental similarity and climate anomaly levels under different gas emission scenarios (ssp126, ssp245, ssp370, ssp585) to reveal the environmental change trends for *A. officinarum* in China in the future (**Figure 7**). The results show that by 2050s, the mean values across the scenarios are quite close (around 10.60-11.00), indicating high multivariate environmental similarity and relatively small climate differences. However, by 2090s, the mean value in the high-emission scenario (ssp585) significantly drops to 7.53, suggesting an increase in environmental variability, with conditions in different regions significantly deviating from the training data of model, leading to reduced prediction reliability. The spatial distribution maps further show that under high-emission scenarios (ssp370 and ssp585), particularly in the 2081-2100 period predictions, the degree of climate anomalies increases, indicating a heightened risk of future extreme climate events. In contrast, low-emission scenarios (ssp126 and ssp245) exhibit higher environmental similarity and more moderate impacts from climate change, with a lower risk of climate anomalies.

**Figure 8:**
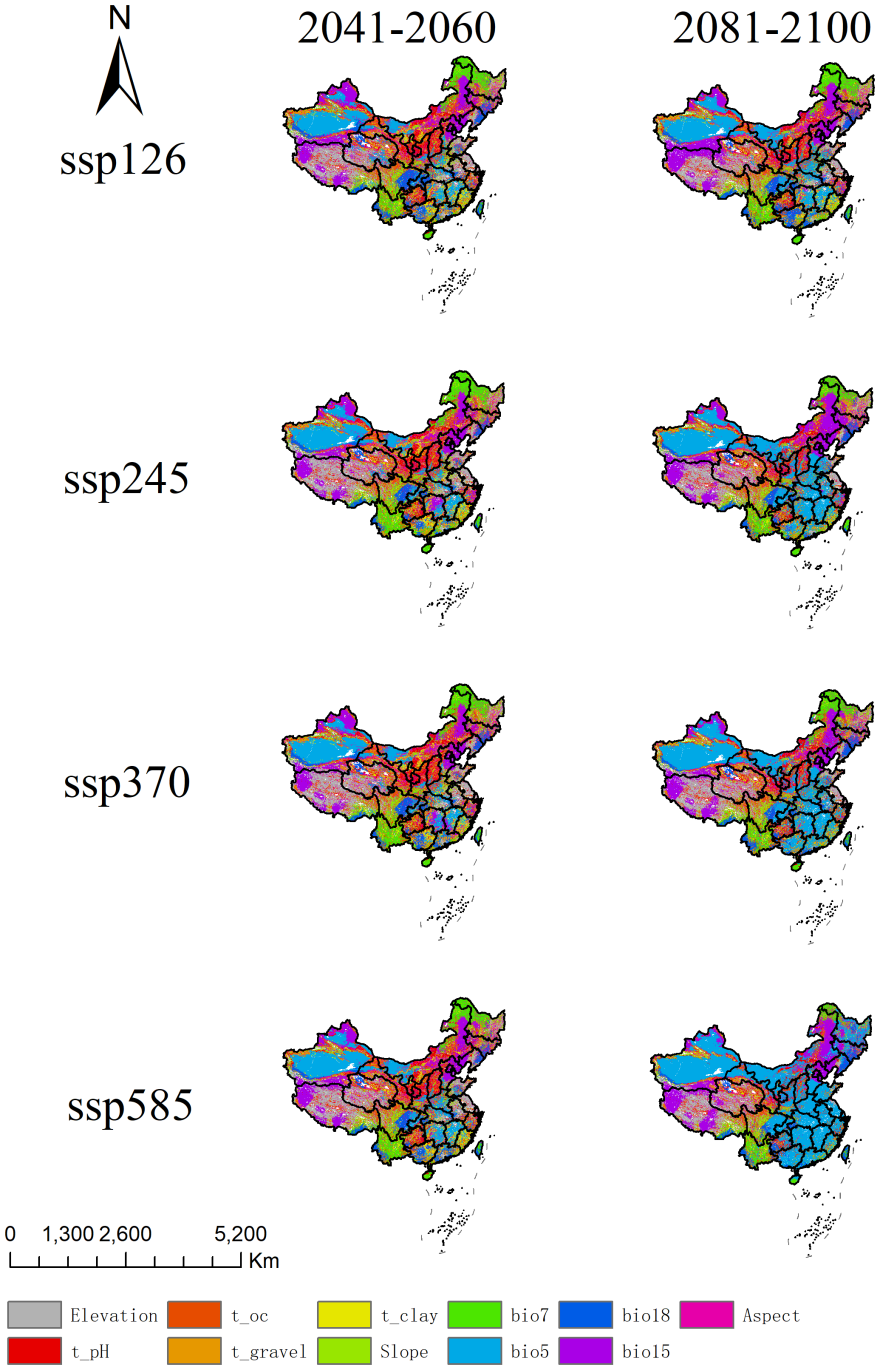
Analysis of most dissimilar variables for *A. officinarum* suitable habitats under different climate scenarios.

Based on the MoD results, this study analyzed the primary driving factors of environmental changes in China under different emission scenarios (ssp126, ssp245, ssp370, ssp585) (**Figure 8**). The results show that in high-emission scenarios (ssp370 and ssp585), temperature-related factors (e.g., bio5 and bio15) dominate, especially in the 2081-2100 period, where the trend of increasing temperatures is significant, affecting most of the suitable habitat areas. Precipitation factors (e.g., bio18) also show noticeable changes. The influence of topographic and soil factors (e.g., Elevation, pH, Slope) is more scattered. In contrast, under low-emission scenarios (ssp126 and ssp245), changes in temperature and precipitation are smaller, and environmental conditions remain more stable.

### 3.7 Migration trends of the center point of *A. officinarum* suitable habitat under climate change

**Figure 9:**
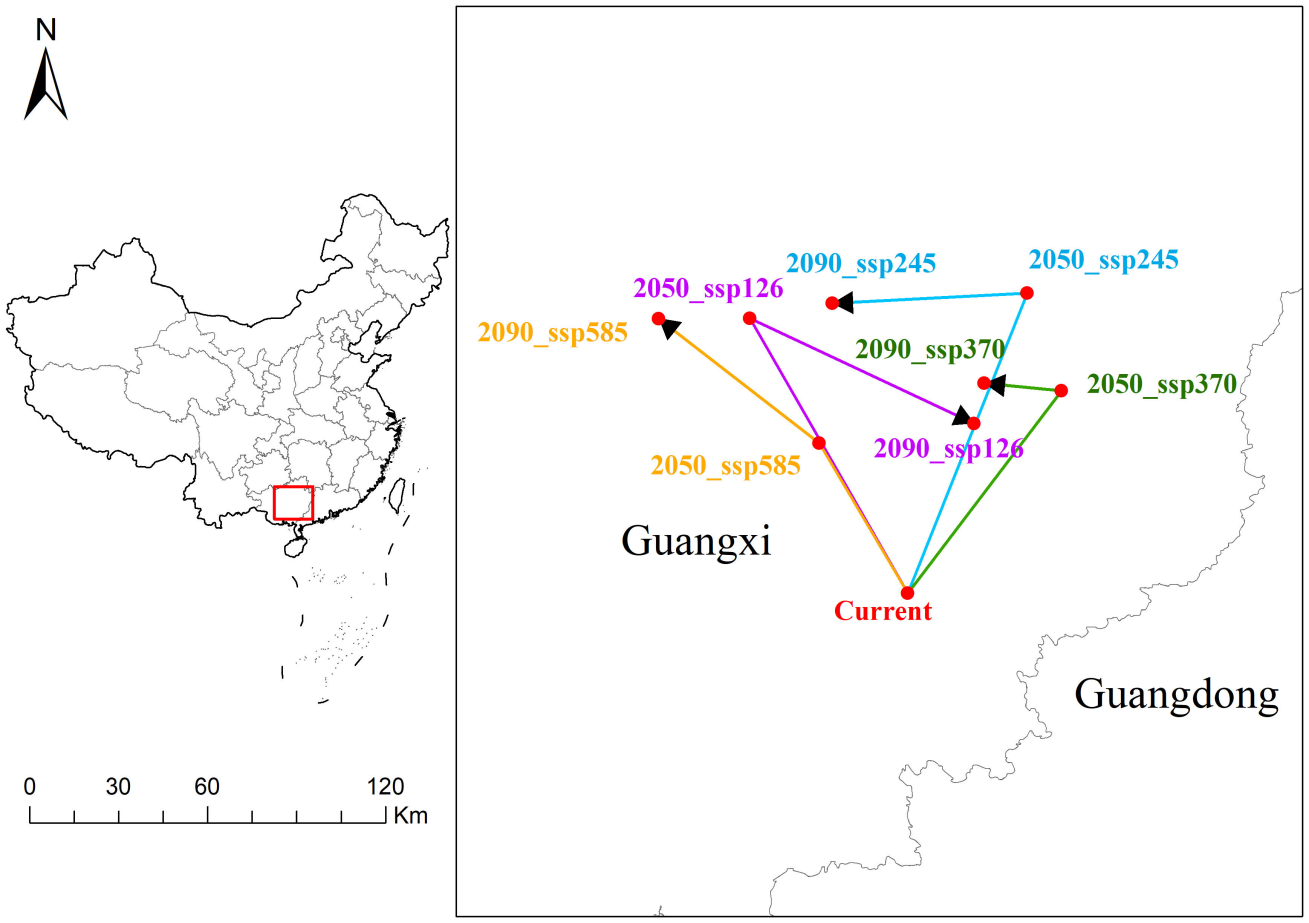
Migration path of *A. officinarum* suitable habitat centers under current and future climate scenarios.

The spatial distribution of the current point and the points under different emission scenarios in Guangxi and Guangdong can be visually observed in the **Figure 9**. By connecting the current point with future points (2050s and 2090s) under different emission scenarios (ssp126, ssp245, ssp370, ssp585), the spatial relationships are illustrated. In the ssp126 scenario, 2050_ssp126 and 2090_ssp126 are located northwest of the current point. The distance from the current point is 104.45 km in 2050s, decreasing to 60.59 km by 2090s, indicating a significant trend of convergence. In the ssp245 scenario, 2050_ssp245 and 2090_ssp245 are relatively close, located slightly north-northeast of the current point, with minor changes in distance (107.25 km and 100.04 km), suggesting a high level of geographical stability. The ssp370 scenario shows 2050_ssp370 and 2090_ssp370 to the northeast of the current point, with both points maintaining a distance of 82.99 km from the current, demonstrating a high degree of spatial stability. In the ssp585 scenario, 2050_ssp585 and 2090_ssp585 are positioned northwest of the current point, with slight displacement and an overall greater distance, indicating spatial variation.

## 4 Discussion

### 4.1 Maxent model optimization and improvement in predictive accuracy

To minimize error in this study, eight regularization multiplier parameters were set, ranging from 0.5 to 4.0, combined with six feature combinations (L, LQ, H, LQH, LQHP, and LQHPT). These parameter combinations were tested using the ENMeval package in R 4.3.3. Previous studies have rarely combined the ENMeval package (Kass et al., 2021) with the Biomod2 package (Thuiller et al., 2009). Under the default parameter settings (RM=1, FC=LQHPT), the model results showed a delta AICc of 111.06. After optimization, the model parameters were adjusted to RM=4.0 and FC=L, and the delta AICc was reduced to 0. This reduction in delta AICc indicates that the optimized model achieved an optimal balance between model fit and complexity, leading to more accurate species distribution predictions. In fact, the results of this study show that the optimized Maxent model (with parameters FC = L and RM = 4.0) performed the best (**Figure 3**). While the default Maxent parameters generally perform well in terms of model complexity and fit, many researchers have noted that these settings can sometimes lead to overfitting, particularly when multiple factors are incorporated, such as climate, topography, and soil (Li et al., 2020). Overfitting can compromise prediction accuracy and often results in fluctuating environmental response curves that are difficult to interpret. Therefore, the optimized parameters used in this study were crucial in avoiding such issues and improving model performance. By using the optimized Maxent model, overfitting can be effectively reduced, enhancing prediction accuracy, while the response curves become smoother, showing a trend closer to a normal distribution (Yan et al., 2021). Among the eight models tested, the Maxent model provided the best fit to the actual distribution. When comparing the response curves of key environmental variables with different parameter settings, the optimized model displayed considerably more stable curves than the default settings (**Figure 4**). The optimized Maxent model successfully and reliably predicted the potential distribution of *A. officinarum* in China.

### 4.2 The relationship between changes in suitable habitat area for *A. officinarum* and environmental factors under low and high emission scenarios

This study analyzed the expansion and contraction of *A. officinarum* habitats under various climate change scenarios. By comparing low-emission scenarios (ssp126 and ssp245) and high-emission scenarios (ssp370 and ssp585), significant dynamic changes in habitat area were revealed.

Under low-emission scenarios (ssp126 and ssp245), the habitat area for *A. officinarum* exhibited a notable expansion trend. As shown in **Table 3**, the current suitable habitat area is 157.7×10^4^ km². Under the ssp126 scenario, by the 2050s, the suitable habitat expands to 169.7×10^4^ km², reflecting an increase of approximately 7.6%. By the 2081-2100 period, although the habitat area slightly decreases to 150.2×10^4^ km², the overall expansion trend remains evident. The expansion trend is even more pronounced under the ssp245 scenario. By the 2050s, the suitable habitat area increases to 164.7×10^4^ km², an expansion of approximately 4.4%. By the 2090s, the suitable habitat area further increases to 174.6×10^4^ km², a 10.7% expansion. The expansion of suitable habitats for *A. officinarum* is closely related to the combined effects of rising temperatures, improved precipitation, as well as soil and topographic conditions. Specifically, under the ssp126 scenario, the expansion is relatively stable, with moderate climate changes leading to optimized habitat conditions through steady temperature increases and moderate precipitation improvements. In contrast, under the ssp245 scenario, the expansion is more pronounced, reflecting not only significant temperature increases and enhanced precipitation but also the combined influence of soil nutrients and topographic factors, which provide stronger support for the species to expand to higher latitudes and elevations. This trend aligns with recent studies over the past decade on the effects of climate change on plant distribution (Lin et al., 2024). Global warming has provided many plant species with opportunities to migrate northward or expand to higher altitudes (Telwala et al., 2013). Rubenstein et al. noted that climate warming drives plant distribution toward higher latitudes or elevations, particularly increasing habitat suitability in marginal areas (Rubenstein et al., 2023). Under low-emission scenarios, species with strong climate adaptability often see their suitable habitats expand in marginal regions (Tian et al., 2023). The expansion trend of *A. officinarum* habitat observed in this study strongly supports this conclusion.

In contrast, under high-emission scenarios, the suitable habitat for *A. officinarum* significantly contracts. In the ssp370 scenario, the suitable habitat shrinks to 151.86×10^4^ km² by 2050s (a reduction of 3.7%) and further decreases to 147.54×10^4^ km² by 2090s (a reduction of 6.4%). Under the most extreme ssp585 scenario, the habitat shrinks to 126.51×10^4^ km² by 2050s (a 19.8% reduction), with a slight recovery to 169.59×10^4^ km² by 2090s. The contraction of suitable habitats for *A. officinarum* is associated with extreme temperature increases, unstable precipitation patterns, and changes in soil and topographic conditions. In these scenarios, rising temperatures may exceed the species’ optimal range, especially during the warmest months, while enhanced precipitation seasonality and more frequent droughts could disrupt water resource balance, thereby limiting habitat suitability to some extent. Additionally, changes in soil conditions, such as reduced soil organic carbon content, and alterations in slope or topography in certain regions may also pose challenges to habitat sustainability. Currently, the primary cultivation region for *A. officinarum* in China is located in Xuwen, Guangdong. However, with the increasing impacts of high-emission scenarios, cultivation areas may gradually shift northward to adapt to the changing environmental conditions. These results align with findings in various studies. For example, high-emission scenarios suggest that tropical plant habitats may sharply decrease due to rapid temperature increases and more frequent extreme weather events, particularly for species sensitive to climate change (Lei et al., 2024). Extreme heat and drought scenarios threaten the survival of many plants by reducing their habitats (Niggli et al., 2022), consistent with the contraction trends observed for *A. officinarum* in high-emission scenarios. Additionally, research indicates that under high-emission scenarios, *A. officinarum* habitats may migrate northward, but its original southern habitats will shrink significantly, increasing ecological pressure. Extreme climate conditions pose a major threat to tropical plant habitats (Hollenbeck and Sax, 2024), further supporting the findings of this study.

Under different climate scenarios, the suitable habitat expansion and contraction of *A. officinarum* show significant dynamic changes (**Figure 6**). In low-emission scenarios (such as ssp126 and ssp245), the expansion areas increase substantially, particularly under the ssp245 scenario, where the expansion area reaches 16.76×10^4^ km² by 2081-2100 period. This expansion trend is consistent with recent research on plant distribution expansion. As temperatures have risen in recent years, many plant species have expanded towards higher latitudes or altitudes, providing them with new ecological niches and habitats (Jump and Peñuelas, 2005). Furthermore, studies have also found that climate change offers opportunities for plants to expand their boundaries, especially under relatively mild climate scenarios, where habitat expansion is particularly notable (Mao et al., 2024).However, under high-emission scenarios (such as ssp370 and ssp585), *A. officinarum* habitats undergo significant contraction. According to the data, by 2081-2100 period, the contraction area reaches 12.38×10^4^ km² under the ssp370 scenario, while in the ssp585 scenario, the contraction area is 0.95×10^4^ km². Under high emission scenarios, increased greenhouse gas emissions intensify global temperature rise, leading to more frequent and severe extreme climate events, particularly in tropical regions. These extreme events, such as high temperatures and droughts, directly impact the habitat of *A. officinarum*. High temperatures may exceed the heat tolerance of *A. officinarum*, limiting growth and shortening the flowering period, while drought exacerbates water shortages, threatening habitat sustainability. Additionally, extreme climate events may indirectly cause habitat contraction by affecting other species. The migration trend of the center point of *A. officinarum’s* suitable habitat shows that, under high emission scenarios, its suitable habitat is also shifting northward (**Figure 9**). As temperatures rise and extreme climate events increase, many plant species will face severe habitat loss, particularly in tropical regions (Sentinella et al., 2020). Habitat contraction under high-emission scenarios is a widespread phenomenon, especially under conditions of unstable precipitation patterns and extreme heat, where tropical plants face significant survival pressure (GushChina et al., 2023).

Therefore, although the habitat expansion of *A. officinarum* under low-emission scenarios offers potential opportunities for habitat expansion, the contraction trend under high-emission scenarios suggests that its survival faces severe threats in the future. The response of plants to climate change involves not only temperature variations but also complex ecological factors such as water availability and land use (Sentinella et al., 2020). Thus, to ensure the long-term survival of *A. officinarum*, future conservation strategies need to integrate these factors and prioritize the protection of areas most impacted by climate change.

### 4.3 Impact of climate change revealed by multivariate environmental similarity surface and most dissimilar variable

Through MESS and MoD analysis, the dynamic changes in *A. officinarum* suitable habitats under different climate scenarios clearly reveal the impacts of future climate change. In low-emission scenarios (ssp126, ssp245), the effects of climate change are relatively mild. The MESS analysis results for 2050s and 2090s indicate that environmental similarity remains at a high level, with low climate variability. Under low-emission scenarios, the climate adaptability of biological communities is strong, the frequency of extreme climate events is lower, and there are minimal changes in habitat suitability (Chong, 2014). Seddon et al. also support this view, suggesting that the impact of climate change on ecosystems is relatively small in low-emission scenarios, allowing species to maintain stable habitats (Seddon et al., 2020).

However, under high-emission scenarios (ssp370, ssp585), MESS analysis shows a significant increase in areas with climate anomalies and a substantial rise in environmental variability. The MoD analysis further indicates that temperature-related factors (such as bio5) and precipitation-related factors (such as bio15) will become key drivers affecting *A. officinarum* habitats in the coming decades. This is consistent with the findings of Deb et al., who emphasized that changes in temperature and precipitation patterns will have profound impacts on the distribution of tropical plants, with extreme temperature events likely leading to a significant reduction in species distribution (Deb et al., 2018). Climate warming will alter the survival environment of tropical plants, particularly under high-emission scenarios, where increased climate variability will result in a dramatic reduction in species habitats (Sun et al., 2022).

The risks under high-emission scenarios are evident, particularly in the ssp585 scenario, where MoD analyses indicate that extreme climate conditions will significantly increase in the future. Under high-emission scenarios (such as ssp585), significant changes in key environmental variables can be visually observed through the colors in **Figure 8**, particularly in the areas represented by the blue bio5 and purple bio15, clearly reflecting the intensification of temperature and precipitation changes. Firstly, the color distribution of bio5 under the ssp585 scenario shows a substantial regional expansion, indicating a significant increase in the range and intensity of high temperatures, especially in tropical and subtropical regions. This persistent temperature rise exceeds the ecological tolerance range of *A. officinarum*, thereby inhibiting its growth and survival. Secondly, the distribution of bio15 under the ssp585 scenario also exhibits notable changes, with enhanced precipitation variability in multiple regions. This variability may lead to more frequent droughts and heavy rainfall events, further increasing environmental instability. Based on the regional distribution in the figure, areas with more pronounced changes in bio15 often overlap with regions of high topographic complexity, suggesting that terrain factors such as slope and aspect may also exacerbate variations in precipitation patterns. The instability in temperature and precipitation will have a devastating impact on the distribution of *A. officinarum*. Future extreme climate events are expected to severely affect habitat stability, especially in tropical and subtropical regions (Hollenbeck and Sax, 2024). Therefore, the threat of climate change to *A. officinarum* habitats under high-emission scenarios is especially pronounced based on the MESS and MoD analyses. Future conservation strategies should focus on mitigating the negative impacts of climate variability and strengthening the protection and management of species habitats in the face of climate change.

### 4.4 Migration of *A. officinarum* habitat centers under climate change

Under climate change scenarios, the center points of *A. officinarum* suitable habitats shows significant spatiotemporal shifts, primarily migrating northward or northwestward due to rising temperatures and changing precipitation patterns (**Figure 9**). In low-emission scenarios (such as ssp126 and ssp245), the center points gradually move northwest in 2050s and 2090s, with the distance to the current point shortening, indicating that the impact of climate change on its suitable habitat is relatively small and the overall environment tends to remain stable. Studies have shown that suitable habitats are concentrated in areas with better climate adaptability, which aligns with the findings of Wang, Y. et al. suggesting that species distribution remains relatively stable under low-emission scenarios, with strong adaptive capacity and no large-scale habitat migration (Wang et al., 2023a). However, in high-emission scenarios (such as ssp370 and ssp585), the shifts become more pronounced and complex, driven by extreme events such as droughts and heatwaves, which create greater environmental instability and contribute to more erratic migration patterns. Importantly, the geometric center remains relatively stable in the ssp370 scenario, with the distance from the current points remaining within 82.99 kilometers in both 2050s and 2090s. In some regions, local ecological conditions can mitigate the effects of climate change on species distribution, helping habitats remain stable in the short term (Kosanic et al., 2018). However, under the ssp585 scenario, the center points of *A. officinarum* shows significant displacement, indicating that as extreme climate conditions intensify, its suitable habitat may face greater pressure and a more extensive migration trend. This phenomenon is consistent with the findings of Xu et al., who suggest that tropical and subtropical species will experience a substantial reduction in habitat under high-emission scenarios, particularly with unstable precipitation patterns and significantly rising temperatures (Xu et al., 2023). As climate change progresses, species distribution in biodiversity hotspots may undergo significant changes, with the high instability of habitats affecting survival capabilities of species (Rubenstein et al., 2023).

In summary, the findings of this study highlight the need for targeted conservation strategies based on climate change scenarios. Under low-emission scenarios, habitat expansion provides opportunities for *A. officinarum* to extend its range, particularly in marginal areas at higher latitudes or altitudes, which should be prioritized for protection. Conversely, high-emission scenarios predict significant habitat contraction, particularly in tropical regions, due to rising temperatures and extreme climate events. Urgent conservation measures are needed to mitigate the impacts of these changes, focusing on protecting vulnerable habitats from extreme conditions such as drought and high temperatures. Additionally, as habitats may shift under climate change, conservation efforts should also include establishing migration corridors to facilitate species adaptation to new climatic conditions. For cultivated populations, it is recommended to explore alternative planting sites in higher latitudes or altitudes to preemptively address the impacts of habitat shifts. Furthermore, implementing adaptive agricultural practices, such as selecting drought-resistant varieties and optimizing irrigation systems, could help maintain sustainable cultivation. These strategies are essential to ensure the long-term survival of *A. officinarum* under changing environmental conditions.

## 5 Conclusion

This study provides important insights into the future distribution patterns of *A. officinarum* under different climate change scenarios by utilizing optimized Maxent and Biomod2 models. Under the ssp126 and ssp245 scenarios, the suitable habitat area for *A. officinarum* expands in both periods. By the 2050s, the area increases to 169.7×10^4^ km² under ssp126 and 164.7×10^4^ km² under ssp245. By the 2090s, the area changes to 150.2×10^4^ km² under ssp126 and further increases to 174.6×10^4^ km² under ssp245, highlighting a clear expansion trend, especially under ssp245. These projections indicate that under low-emission scenarios (ssp126 and ssp245), the suitable habitat for *A. officinarum* will continue to expand, with stable and gradual shifts in its geographic center towards the northwest. This trend suggests that the species can adapt well to milder climate changes, with potential opportunities for range expansion. The findings align with research showing that plants with high climate adaptability can benefit from expanded suitable habitats in moderate climate change conditions.

Conversely, under high-emission scenarios (ssp370 and ssp585), *A. officinarum* faces significant habitat contraction, with its geographic center undergoing larger migrations and instability, particularly in the ssp585 scenario. This contraction is driven by increased climate variability, accompanied by rising temperatures and greater instability in precipitation patterns, as reflected in significant changes in key factors such as bio5 (maximum temperature of the warmest month) and bio15 (precipitation seasonality). These changes have contributed to the occurrence of extreme conditions such as high temperatures and droughts, which have become major limiting factors. These findings highlight the substantial risks that *A. officinarum* and other tropical species may face in the future, with habitat loss and ecological pressure increasing in regions vulnerable to extreme climate events.

Overall, while low-emission scenarios offer potential opportunities for habitat expansion, high-emission scenarios present a clear threat to the long-term survival of *A. officinarum*. Therefore, future conservation strategies should prioritize addressing the impacts of climate change, with particular emphasis on protecting areas most vulnerable under high-emission scenarios to ensure the long-term adaptability and survival of *A. officinarum*.

## Data Availability

The raw data supporting the conclusions of this article will be made available by the authors, without undue reservation.
